# Bigger and Better: How Pfizer Redefined Erectile Dysfunction

**DOI:** 10.1371/journal.pmed.0030132

**Published:** 2006-04-11

**Authors:** Joel Lexchin

## Abstract

Lexchin examines how Pfizer transformed sildenafil from an effective product for erectile dysfunction due to illness or injury into a drug that healthy men can use to enhance their erections.


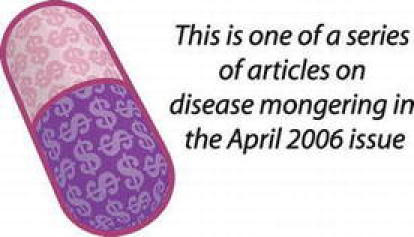


In the pursuit of profits, pharmaceutical companies are continuously looking to expand the market for their products. This article examines how Pfizer transformed Viagra from an effective product for erectile dysfunction (ED) due to medical problems, such as diabetes and spinal cord damage, into a drug that “normal” men can use to enhance their ability to achieve an erection and to maintain it (in a “harder” state) for a longer period of time.

## The Rise of Lifestyle Drugs

An important emerging issue in health care is the availability of medications to treat what until recently have been regarded as the natural results of aging or as part of the normal range of human emotions. Thus, we now see treatments widely advertised for male pattern baldness and shyness. Deviating even further, drug therapy is moving out of treating diseases to providing enhancements to what had hitherto been seen as normal functioning. This evolution in the use of medications has introduced dilemmas and controversies about what are legitimate conditions and treatments for those concerned with prescription medications: is any deviation from normality fair game for treatment? What about people who have nothing medically wrong with them, but just want to feel better? Who will pay for these therapies, and what are the implications for the way we use health-care resources?

Medications that embody these controversies are generally referred to as lifestyle drugs and perhaps the best known of these is sildenafil citrate (Viagra) This article will examine the strategies used by Pfizer, the maker of Viagra, to ensure that the drug was seen as legitimate therapy for almost any man. Pfizer took steps to make sure that Viagra was not relegated to a niche role of just treating men who had ED due to organic causes, such as diabetes or prostate surgery.

There is no doubt that Viagra is an effective and quite safe drug in treating ED secondary to these causes, although a systematic review of the evidence found that the drug probably only results in successful intercourse 50%–60% of the time [ 1]. Had Viagra been confined to use only in cases of ED secondary to organic causes, the drug would probably have been a modest success for Pfizer. In order to grow the market, Pfizer had to make Viagra the treatment of choice for a much wider population of men. The perceived prevalence of ED needed to be expanded. The impression had to be created that ED was of significant concern to many, perhaps even most, men or at least those over 40 years of age. The criterion of success for treating ED had to be redefined. And finally, Viagra had to be seen as an important treatment option for men with any degree of ED, including rare or transitory failures to achieve or maintain erections.

## Redefining the Prevalence of ED and Its Psychological Effects

On its Web site, Pfizer states that “in fact, more than half of all men over 40 have difficulties getting or maintaining an erection” ( http://www.viagra.com/ed/index.asp). The Web site does not give a reference to support this statement. One possible source of support for this statement is the Massachusetts Male Aging Study (MMAS), a community-based, random sample observational survey of men aged 40 to 70 years old conducted from 1987 to 1989 in cities and towns near Boston, Massachusetts [ 2]. The authors of the study extrapolated the results to argue that 52% of the entire male population in the United States between the ages of 40 and 70 suffer from ED. The authors stated: “In the MMAS sample the prevalence of impotence of all degrees was estimated at 52%. Projection of these results to 1990 population data would suggest that impotence affects 18 million American men 40 to 70 years old” [ 2]. However, the MMAS figures must be viewed with a number of caveats.

First, there were actually two different groups of men in this study. The first, and larger, group answered a series of nine questions about sexual activity. The second, and much smaller, group answered the same nine questions, plus an additional question to self-rate themselves as not impotent, minimally impotent, moderately impotent, or completely impotent. The answers to this final question by the men in the second group were then applied to the first group to derive the percent in the various classes of potency. The authors do not provide any information about whether the two groups were similar, and there are reasons to think that differences may exist between the groups. The first group was randomly selected from towns and cities in the Boston Standard Metropolitan Statistical Area [ 3], while the second group was made up of men presenting to a university center urology clinic [ 2].

Even if the scores from one group can be transferred to the other, the 52% figure is still deceptive because it doesn't differentiate ED by age. In the MMAS, 40% of 40-year-old men had ED, including 17% who were only minimally impotent, whereas 67% of 70 year olds were impotent. Moreover, not all studies are in agreement with these figures. Analysis of data from the US National Health and Social Life Survey indicates that among men 50–59 years old, 18% complained of trouble achieving or maintaining an erection during the past year [ 4]. A survey in the Netherlands found that only 1% of men 50–65 years of age had a complete inability to achieve an erection, and it was only in men aged 70–78 years that the rate of ED was similar to that in the MMAS [ 5]. Out of 13 studies on the prevalence of ED that were published until June 1998, the MMAS results were among the highest [ 1]. Thus, Pfizer's statement that “more than half of all men over 40 have difficulties getting or maintaining an erection” does not reflect the large variation in the prevalence of ED found in different studies.

The MMAS found a strong association between ED and psychological factors, including “depression, low levels of dominance, and anger either expressed outward or directed inward.” The authors suggested that psychological symptoms might be a cause of ED, but these symptoms could also be an effect of ED (they wrote that “a man who has experienced a recent pattern of ED may be expected to be anxious, depressed and lacking self-esteem and self-confidence”) [ 2]. While not to deny that there is an association between ED and psychological symptoms, once again the MMAS may be an outlier. In the Dutch study previously mentioned, only one-third of all men and only 20% of men over the age of 70 with significant ED had major psychological concerns. Furthermore, in sexually active men, 17%–28% had no normal erections, indicating that full erectile function is not essential for sexual functioning [ 5]. Only 20% of Japanese men 40 to 79 years of age reported more than little worry and concern about sexual functioning, suggesting that perceptions of elderly male sexual function and its impact on health-related quality of life may differ among cultures and ethnic groups with differing values [ 6].

On its Web site, Pfizer states: “VIAGRA can work for you. In fact, studies show that VIAGRA works for more than 80% of men with ED taking VIAGRA 100 mg versus 24% of men taking a sugar pill” ( http://www.viagra.com/whyViagra/highlyEffective.asp). The 80% success rate that Pfizer quotes for Viagra is important, though not critical, to being able to promote its use to a wide variety of men. But that number is qualified on the Pfizer Web site as the number who experience improved erections ( http://www.viagra.com/consumer/aboutViagra/index.asp). It is open to speculation whether the goal of most men is improved erections, or successful intercourse and the achievement of an orgasm. In most studies on Viagra, a 50%–60% rate of successful intercourse is recorded (in the dose titration studies reviewed in [ 1] for patients taking placebo, up to 25% of attempts at intercourse were successful compared with 50%–60% for patients taking Viagra 25–100 mg). This 50%–60% rate is far short of the “more than 80% of men” that Pfizer trumpets.

## Viagra for Any Degree of ED

To make Viagra into a lifestyle drug, Pfizer needs to convince men that it is the first choice for therapy for any degree of ED, whatever the genesis of the problem. However, drug therapy may not always be the most appropriate treatment option. The National Health and Social Life Survey data indicate that emotional and stress-related problems such as a deteriorating social and economic position generate elevated risk of experiencing sexual difficulties. In these cases, Viagra may be less important than counseling or help in finding a new job. These possibilities are never mentioned on the Viagra Web site. Here is a sample of the questions and answers on the “About ED” portion of the Web site:

Question: “I don't have ED because the problem doesn't happen often. Does this mean that VIAGRA is not for me?”

Answer: “Even if erection problems happen only once in a while, VIAGRA can help. You should know that most men with ED only experience problems some of the time. In one study, VIAGRA helped 87% of men with mild-to-moderate ED have better erections versus 36% of men taking a sugar pill” ( http://www.viagra.com/faqs/faqs2.asp).

In case the message is missed, there is a couple on the Web page where the man looks to be in his mid-to-late 30s. Pfizer reinforces its message with direct-to-consumer magazine ads, such as one featuring a virile looking man around 40 saying, “A lot of guys have occasional erection problems. I chose not to accept mine and asked about Viagra.”

Drug companies have identified lifestyle drugs as a “growth market.”

The initial television ads in the US for Viagra used an aging Bob Dole (born 1923) as a spokesman, a 1996 Republican presidential candidate. Since then, Pfizer has refocused its advertising campaign to match the lifestyle message on its Web site. There is now advertising of Viagra at NASCAR races, and Pfizer hired 39-year-old Rafael Palmeiro, a former Texas Ranger baseball player as a spokesman ( Figure 1) [ 7]. Pfizer teamed up with *Sports Illustrated* magazine to create the Sportsman of the Year Trivia Game ( http://www.viagra.com/sports/index.asp). Between 1999 and 2001, Pfizer spent over US$303 million in direct-to-consumer advertising to get its message about Viagra to men [ 8–10]. Besides the large promotion budget, Pfizer has also paid a number of doctors to act as “consultants,” delivering public lectures and appearing in the mass media to expound on ED and Viagra [ 11]

**Figure 1:**
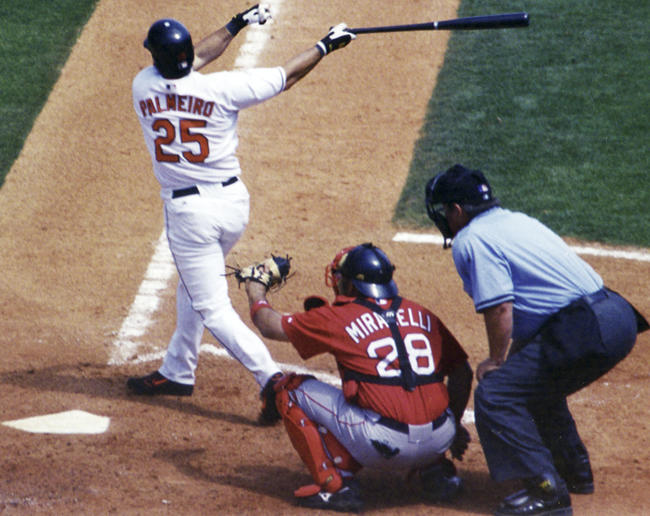
Pfizer Hired 39-Year-Old Baseball Player Rafael Palmeiro as a Spokesman for Viagra

Pfizer denies that it is targeting younger men or that it is positioning Viagra as a lifestyle drug. Mariann Caprino, a spokeswoman for the company, is quoted in the *New York Times* as saying, “Have we gone out and given our advertising agency instructions to speak to this young population? No, we haven't” [ 7]. But the message from the pictures on the Web site, in magazine ads, and from people like Rafael Palmiero is that everyone, whatever their age, at one time or another, can use a little enhancement; and any deviation from perfect erectile function means a diagnosis of ED and treatment with Viagra. Increasingly, the age profile of men using Viagra reflects the younger audience that Pfizer denies it is targeting. Between 1998 and 2002 the group showing the largest increase in Viagra use was men between the ages of 18 and 45, and only one-third of these men had a possible etiologic reason for needing Viagra [ 12].

## Economic and Social Implications of the Expanding Market for Lifestyle Drugs

Drug companies have identified lifestyle drugs as a “growth market.” The problems that they are designed to treat are easily self-diagnosed—we can all see if we are bald or fat—and as the baby boomers age, the population looking to these drugs will continue to grow. Drug companies, driven by profit, go where the money is.

Because of the potential size of the market for Viagra, paying for it in unlimited quantities will be very expensive. Viagra may only be the tip of the iceberg. If we believe the prophets of technology, soon there will be drugs for memory enhancement and the possibility of genetic manipulation to make us taller or to keep a full head of hair. Here we come back to the enhancement debate. Do we accept our limitations with grace, or is it legitimate to seek technological solutions for them? In one corner is the view of health as freedom from disease, where “the central purpose of health care is to maintain, restore, or compensate for the restricted opportunity and loss of function caused by disease and disability” [ 13]. In this model, a just medical system would not cover treatments and interventions that aim to enhance abilities not affected by disease and disability. Opposing this is an expansionist definition, such as the one offered by the World Health Organization, where health is “a state of complete physical, mental and social well-being” ( http://www.who.int/about/definition/en/print.html). If we accept this view, then are we not obliged to provide for people who want to enhance themselves so that they can achieve mental and social well-being?

This debate is further complicated because there is not an equal balance in how we look at the options of accepting limitations and seeking enhancement. In a market-driven world, the money is in promoting enhancements, not in accepting limitations. The ad featuring the man who chooses not to accept even occasional erection problems is one example of how commercial pressures bias the debate [ 14].

Because of the possibility that large numbers of men would request Viagra from their doctors, getting insurance companies to pay for Viagra presented Pfizer with special problems. Early on, Kaiser Permanente refused to cover Viagra for its 9 million members because of costs expected to be in the range of US$100 million per year [ 11]. According to one interpretation, reactions from insurers such as Kaiser Permanente were the reason that Pfizer put in place a US$35 million campaign to change insurers' decisions [ 11]. Another goal of Pfizer's campaign was to make ED an acceptable topic for public discourse, in order to remove the stigma attached to it and increase the possibility that third parties would provide coverage.

## Conclusion

Viagra presents a microcosm of the debate surrounding drugs that enhance lifestyle choices. The drug is effective and safe for people with medical problems warranting treatment, but it also can be used by a much wider population. The company that manufactures the drug, recognizing that the potential market is huge, has aggressively targeted that much larger community. Pfizer's well-financed campaign was aimed at raising awareness of the problem of ED, while at the same time narrowing the treatment possibilities to just a single option: medication. Having succeeded in turning Viagra into a consumer product, Pfizer then turned its attention to payers in order to reap the benefits of the expanded market.

Ultimately, there must be a debate about how limited resources for health care should be spent and who should make those decisions. Are men who seek to enhance their normal sexual function “worthy” enough to have their treatment paid for? If we pay for drugs and other procedures that enhance lifestyles, then other treatments either may not get funded at all or may become inadequately funded. Who will get the lifestyle drugs? Everybody who wants them? And do they get an unlimited supply? As the number of enhancement treatments grows, the scenario surrounding Viagra will become all too familiar with other drugs. Now is the time to start preparing for how we will deal with the inevitable explosion of drugs and other interventions that can make us “better than well” [ 16].

## 

**Figure:**
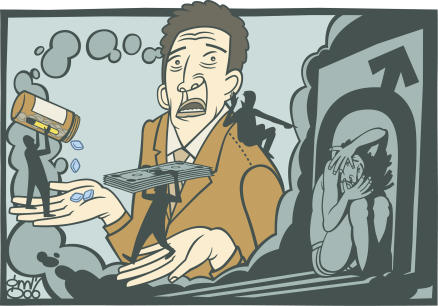
(Illustration: Giovanni Maki)

## Abbreviations

ED: erectile dysfunction
MMAS: Massachusetts Male Aging Study
